# c-MET as a Potential Therapeutic Target in Ovarian Clear Cell Carcinoma

**DOI:** 10.1038/srep38502

**Published:** 2016-12-05

**Authors:** Ha-Jeong Kim, Aera Yoon, Ji-Yoon Ryu, Young-Jae Cho, Jung-Joo Choi, Sang Yong Song, Heejin Bang, Ji Soo Lee, William Chi Cho, Chel Hun Choi, Jeong-Won Lee, Byoung-Gie Kim, Duk-Soo Bae

**Affiliations:** 1 Department of Obstetrics and Gynecology, Institute of Wonkwang Medical Science, College of Medicine, Wonkwang University, Iksan, Korea; 2 Department of Obstetrics and Gynecology, Samsung Medical Center, Sungkyunkwan University School of Medicine, Seoul, Korea; 3 Department of Pathology and Translational Genomics, Samsung Medical Center, Sungkyunkwan University School of Medicine, Seoul, Korea; 4 Health promotion center Samsung Medical Center, Sungkyunkwan University School of Medicine, Seoul, Korea; 5 Department of Clinical Oncology, Queen Elizabeth Hospital, Kowloon, Hong Kong; 6 Institute for Refractory Cancer Research, Samsung Medical Center, Seoul, Korea; 7 Samsung Advanced Institute for Health Sciences & Technology, Sungkyunkwan University School of Medicine, Seoul, Korea

## Abstract

In this study, we investigated the therapeutic effects of c-MET inhibition in ovarian clear cell carcinoma (OCCC). Expression levels of c-MET in the epithelial ovarian cancers (EOCs) and normal ovarian tissues were evaluated using real-time PCR. To test the effects of c-MET inhibitors in OCCC cell lines, we performed MTT and apoptosis assays. We used Western blots to evaluate the expression of c-MET and its down-stream pathway. *In vivo* experiments were performed to test the effects of c-MET inhibitor on tumor growth in orthotopic mouse xenografts of OCCC cell line RMG1 and a patient-derived tumor xenograft (PDX) model of OCCC. c-MET expression was significantly greater in OCCCs compared with serous carcinomas and normal ovarian tissues (p < 0.001). In *in vitro* study, inhibition of c-MET using c-MET inhibitors (SU11274 or crizotinib) significantly decreased the proliferation, and increased the apoptosis of OCCC cells. SU11274 decreased expression of the p-c-MET proteins and blocked the phosphorylation of down-stream proteins Akt and Erk. Furthermore, SU11274 treatment significantly decreased the *in vivo* tumor weight in xenograft models of RMG1 cell and a PDX model for OCCC compared to control (p = 0.004 and p = 0.009, respectively).

Epithelial ovarian cancer (EOC) is one of the leading causes of cancer-related deaths in women, and the most lethal gynecologic malignancy^1^. Recently, there has been increasing recognition that EOC is a highly heterogeneous disease with diverse clinical features and biologic origins^2^. Ovarian clear cell carcinoma (OCCC) is a rare histological type, accounts for 5–15% of all EOCs^3^. Compared with other EOC subtypes, especially, high-grade serous, patients with OCCC have high chemoresistance, high recurrence rate, and poorer clinical outcome in advanced or recurrent settings^4^,^5^,^6^. Therefore the molecular mediators that contribute to progression and metastasis of OCCCs will allow potential therapeutic targets for improved their prognosis.

c-MET is a receptor tyrosine kinase with a high-affinity ligand, hepatocyte growth factor/scatter factor (HGF/SF). In tumor, deregulation of c-MET activity can trigger important cellular processes including to cell proliferation, invasion, survival, and angiogenesis^7^,^8^,^9^. The c-MET/HGF axis also inhibits apoptosis of cancer cells and confers resistance to cell death by conventional chemotherapy^8^. Several studies have described the association between c-MET activation with adverse clinical outcomes in lung, breast, stomach, kidney and head & neck cancer^10^,^11^,^12^,^13^. Accordingly, various c-MET inhibitors have been recently suggested as potential anticancer agents in several cancers^14^.

In EOC, overexpression of c-MET is found in about 7% to 27%^15^,^16^,^17^,^18^ and it is associated with ovarian cancer progression and adverse outcomes^17^,^18^. Recently, study on OCCC reported that c-MET amplification rate was 37.0% and correlated with worse survival^19^. Although several *in vitro* and *in vivo* studies reported that inhibition of c-MET using small interfering RNA and small molecule inhibitors reduced EOC growth and metastasis^16^,^17^,^20^,^21^, the therapeutic effects of c-MET inhibitors in patients with OCCC have seldom been addressed. In the present study, we investigated the effects of c-MET inhibitors in OCCCs with *in vivo* as well as *in vitro* experiments including an orthotopic mouse model using an established cell line (RMG1) and a patient-derived tumor xenograft (PDX) model.

## Results

### Expression of c-MET in human ovarian tissues and cell lines

**Figure 1:**
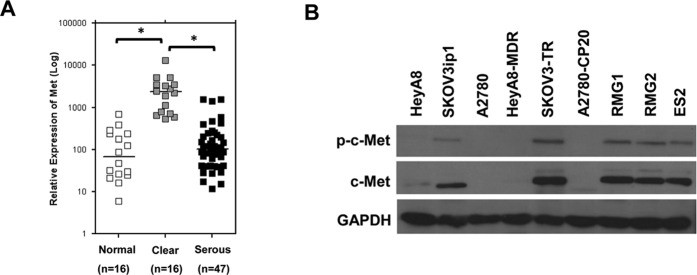
(**A**) Real-time PCR analysis of c-MET expression in human ovarian tissue. Expression of c-MET was significantly higher in ovarian clear cell carcinoma (OCCC) tissues compared with serous carcinoma and normal ovarian tissues (*p < 0.001). (**B**) Expression of c-MET in ovarian cancer cell lines measured using Western blot. Strips corresponding to each of the proteins shown are cropped from different blots run under the same experimental conditions. The original blots were attached as Supplementary Figure 1.

c-MET expression was estimated in human ovarian tissues, including 16 normal ovarian, 47 serous carcinoma and 16 OCCC tissues. The expression level of c-MET was significantly higher in OCCC tissue compared with normal ovarian tissue or serous carcinoma tissue (Fig. 1A, both p < 0.001). We evaluated expression of c-MET and phosphorylated c-MET (p-c-MET) in ovarian cancer cell lines using Western blot. Of the non-OCCC cell lines (HeyA8, SKOV2ip1, A2780, HeyA8-MDR, SKOV3-TR, A2780-CP20), SKOV3ip1 and SKOV3-TR expressed high levels of c-MET protein and p-c-MET. Of note, c-MET protein and p-c-MET were strongly expressed in all OCCC cell lines including RMG1, RMG2 and ES2 cells (Fig. 1B).

### c-MET inhibitors significantly affect cell survival and apoptosis in OCCC cells

**Figure 2:**
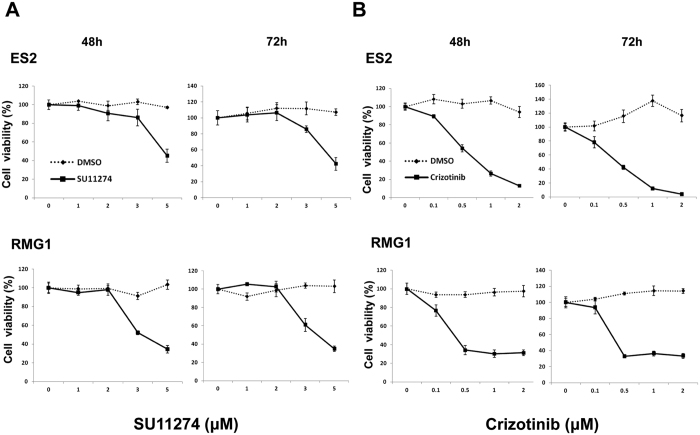
Cell viabilities as assessed by MTT assay were notably reduced by treatment with c-MET inhibitors (**A** SU11274, **B** crizotinib) for up to 72 hours compared with controls in ES2 and RMG1 cells (error bar represents s.e.m.).

**Figure 3:**
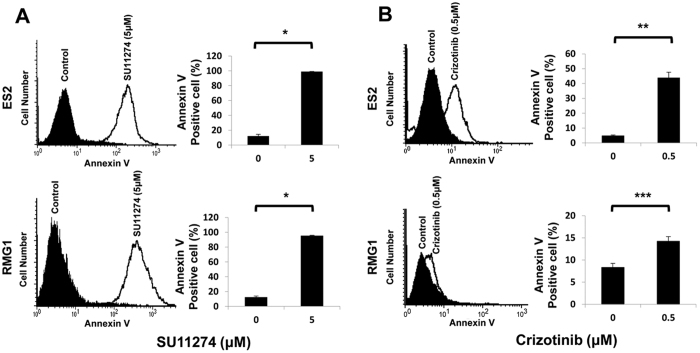
c-MET inhibitors significantly increased cellular apoptosis in OCCC cell lines ES2 and RMG1. Cells treated with (**A**) SU11274 or (**B**) crizotinib showed significantly more apoptosis as assessed by FACS analysis (error bar represents s.e.m. *p < 0.001, **p < 0.010, ***p < 0.050).

We used two kinds of c-MET inhibitors to block the endogenous activity of c-MET in OCCC cells. SU11274 is a selective small molecule c-MET inhibitor, and crizotinib (also known as PF-2341066) is a multikinase inhibitor with known action against c-MET, anaplastic lymphoma kinase (ALK) and c-ros oncogene 1 (ROS1). In the MTT assay, SU11274 and crizotinib significantly reduced cell viability in a dose- dependent manner in both OCCC cell lines, including ES2 and RMG1 (Fig. 2A and B, respectively). In addition, significant increases in apoptosis were seen in ES2 and RMG 1 cells treated with SU11274 compared with controls (Fig. 3A, both p < 0.001) and crizotinib also showed similar effects (Fig. 3B, p = 0.003 and p = 0.030, respectively). To further confirm whether c-MET/p-c-MET expression is associated with the sensitivity of EOC cells to c-MET inhibitors, we performed MTT assay in SKOV3ip1 with low c-MET protein expression and HeyA8-MDR without c-MET protein expression (Fig. 1B). The SU11274 treatment was able to reduce cell viability in SKOV3ip1 but not in HeyA8-MDR cells (Supplementary Figure 4A). However, the effect of SU11274 on cell viability inhibition was greater in OCCC cells with high c-MET expression than in SKOV3ip1 or HeyA8-MDR cells with low c-MET expression. These results suggest that c-MET inhibitor is more effective in OCCC cells that contain high c-MET expression. Because SU11274 selectively inhibited c-MET tyrosine kinase and was more effective at inhibiting cell survival compared to crizotinib (Supplementary Figure 3, and Supplementary Figure 4B), we performed all further *in vitro* and *in vivo* experiments with SU11274.

### SU11274 decreases the expression of phosphorylated downstream proteins of the c-MET signaling pathway

**Figure 4:**
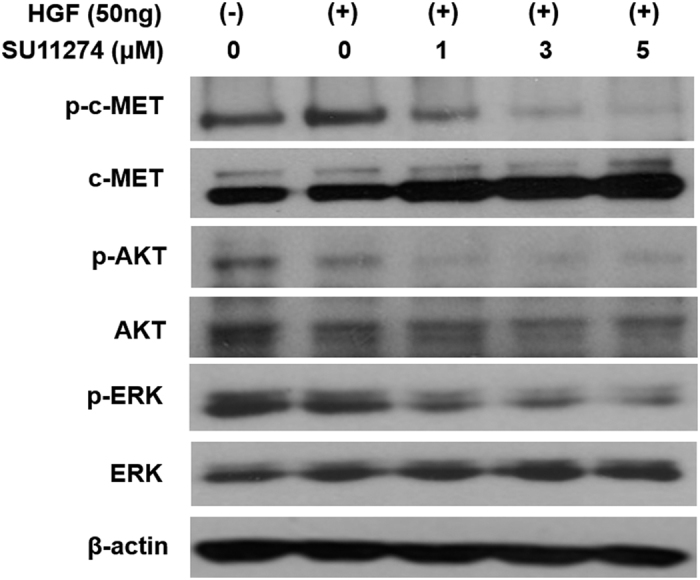
SU11274 inhibited the expression of c-MET and downstream signaling proteins in ES2 cells. Based on Western blots, phospho-c-MET and phosphorylation of downstream signaling proteins including p-Akt and p-Erk were decreased by treatment with SU11274 in a dose-dependent manner. Strips corresponding to each of the proteins shown are cropped from different blots run under the same experimental conditions. The original blots were attached as Supplementary Figure 2.

To ascertain whether the expression of downstream proteins in the c-MET signaling pathway would be affected by a c-MET inhibitor, OCCC cells were stimulated with 25 ng/mL HGF for 20 mins. We examined the effects of SU11274 on phosphorylation of c-MET and the downstream signaling effectors, Akt and Erk. ES2 cells were pretreated with the indicated dose of SU11274 before stimulation with 25 ng/mL HGF for 20 mins. Treatment with SU11274 effectively decreased expression of the p-c-MET proteins and blocked phosphorylation of Akt and Erk, with inhibition of the c-MET downstream proteins in a dose-dependent manner (Fig. 4). Phosphorylation of c-MET was inhibited completely at a concentration of 5 μM SU11274, and phosphorylation of downstream proteins Akt and Erk was diminished.

### SU11274 significantly inhibits tumor growth in an orthotopic EOC mouse model using an established OCCC cell (RMG1)

**Figure 5:**
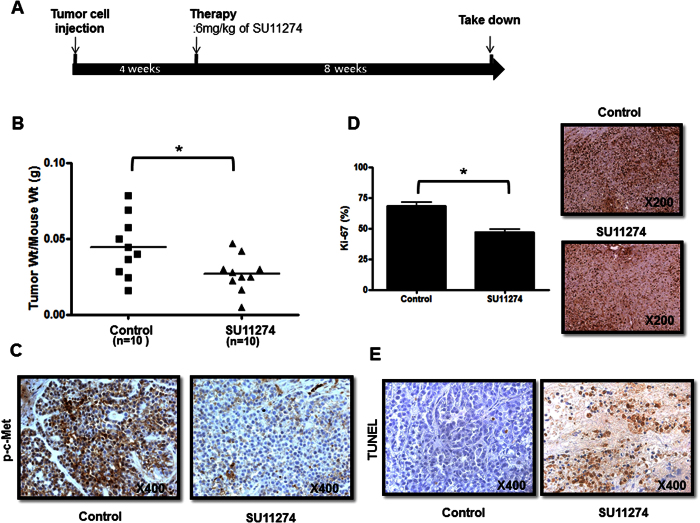
SU11274 treatment significantly reduced tumor growth in an orthotopic OCCC mouse model with RMG1. (**A**) Experimental outline for orthotopic OCCC mouse model. (**B**) The SU11274-treated group (*n* = 10) had significantly lower tumor weights compared with the PBS-injected control group (*n* = 10). (**C**) The SU11274-treated group showed reduced phospho-c-MET expression compared with the control group. (**D**) Tumor cell proliferation as assessed by Ki67 immunohistochemistry in harvested tumor tissues was significantly lower in the SU11274-treated group (*n* = 5). (**E**) TUNEL assay indicated significantly higher apoptosis in the SU11274-treated group (error bar represents s.e.m., *p < 0.010).

An orthotopic model was established in nude mice by intraperitoneal injection (IP) of RMG1 cells (*n* = 10)^22^. We injected SU11274 6 mg/kg IP into the mice every two days starting at day 7 after the injection of cancer cells^23^. The injection of SU11274 resulted in a significantly lower tumor weight compared with controls injected with phosphate buffered saline (PBS) (Fig. 5B, p = 0.004). The expression of p-c-MET protein in harvested tumor tissues was lower in the SU11274 treatment group compared with controls (Fig. 5C). Positive staining for Ki-67 was significantly lower in the SU11274-treated group when we looked for tumor cell proliferation using immunohistochemistry in harvested tumor tissues (Fig. 5D, p = 0.009). TUNEL assay of harvested frozen tissues showed definitively higher apoptosis in the SU11274-treated group compared with controls (Fig. 5E). Moreover, the SU11274-treated group clearly showed a significant increase of caspase-3 expression (Supplementary Figure 6A, p = 0.049).

### SU11274 significantly inhibits tumor growth in a PDX model of OCCC

**Figure 6:**
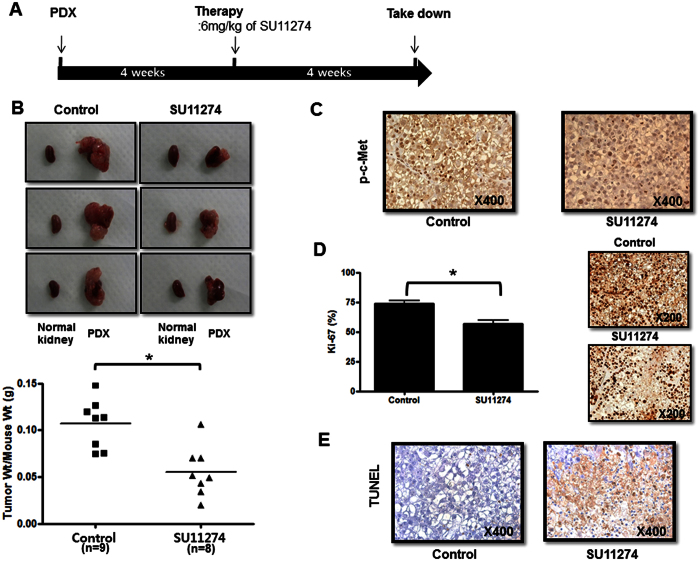
The anti-tumor effects of SU11274 were examined in OCCC patient-derived xenograft (PDX) models. (**A**) Experimental outline for PDX model. (**B**) Tumor weight significantly decreased in the SU11274-treated group (*n* = 9) compared with the PBS-injected control group (*n* = 8). In each picture, the small piece on the left is normal kidney (no tumor transplanted), and the large one on the right is the developed PDX. (**C**) The SU11274-treated group showed less phospho-c-MET expression than the control group. (**D**) Tumor cell proliferation as assessed by Ki67 immunohistochemistry in harvested tumor tissues was significantly lower in the SU11274-treated group (*n* = 5). (**E**) TUNEL assay showed significantly higher apoptosis in the SU11274-treated group (error bar represents s.e.m., *p < 0.010).

We previously developed a PDX model with subrenal implantation for human EOC^24^. The tumors selected for the PDX model came from a patient with FIGO stage IIIC OCCC who received optimal cytoreductive surgery. The PDX model was treated for 4 weeks with SU11274 at the same dose and interval as used for the RMG1 model starting one month after the implantation of xenograft tissues (passage 5). SU11274 significantly inhibited tumor growth in this model (Fig. 6B, p = 0.003). Moreover, we found decreased levels of p-c-MET in harvested cancer tissues compared with controls (Fig. 6C). Ki-67-positive staining was significantly lower in the SU11274-treated group (Fig. 6D, p = 0.001), and the effect of SU11274 in increasing cellular apoptosis was also confirmed in this model (Fig. 6E, and Supplementary Figure 6B, p = 0.026).

## Discussion

In OCCC, previous studies found that MET amplification was observed in >20% of cases and that OCCC with MET amplification exhibited a poor prognosis^19^,^25^. Additionally, Yamashita *et al*. reported that MET knockdown by short-hairpin RNA (shRNA) resulted in a decline of cell viability in MET-amplified OCCC cell lines due to increased apoptosis and senescence, suggesting that the MET signaling pathway plays an important role in OCCC carcinogenesis^19^. Recently, Wang *et al*. reported that c-MET overexpression was associated with chemoresistance and worse prognosis in 86 OCCC patients. Collectively, these data indicate that inhibition of c-MET is a novel strategy for the clinical management of OCCC patients^26^. However, there are few published reports of the effects of c-MET inhibitor on OCCC.

In this study, we found that c-MET expression was significantly higher in OCCC, compared with serous carcinoma or normal ovarian tissue. In addition, c-MET inhibitors (SU11274 or crizotinib) significantly reduced cell viability and enhanced apoptosis in OCCC cells. Moreover, in an *in vivo* orthotopic mouse model of OCCC (RMG1), treatment with SU11274 significantly decreased total tumor weight compared with that of control. These results were corroborated in a PDX model derived from a patient with OCCC, in which treatment with SU11274 significantly reduced tumor growth. These findings indicate that targeting c-MET with SU11274 represents a potential strategy for novel therapeutics for treatment of OCCC. To our knowledge, this study, for the first time, demonstrates the direct effect of c-MET inhibition on tumor growth of OCCC *in vivo* as well as *in vitro.*

c-MET overexpression has been noted in various cancers. In a recent large study of 1115 patients with advanced solid cancers, MET amplification was detected in 2.6%. The highest prevalence was observed in renal cell (14%) and adrenocortical tumors (15%) and MET amplification was observed in 4% of ovarian cancers, all of which were serous carcinomas^27^. Yamashita *et al*. have found MET gene amplification in 37% of OCCC cases^19^ and Wang *et al*. have found c-MET overexpression in 41% of OCCC cases^26^. In this study, c-MET overexpression was found in the OCCC tissues by real-time PCR and c-MET protein was clearly increased in OCCC cell lines by Western blot analysis (Fig. 1).

A systematic review from Furlan *et al*. reported that MET signaling is involved in tumorigenesis, such as tumor proliferation, protection from apoptosis, angiogenesis, and motility^28^. c-MET is frequently activated in EOC^15^,^16^,^17^,^18^ and inhibition of c-MET activity significantly inhibits cell proliferation^1^,^9^,^21^ and metastasis^17^,^20^,^21^,^29^ in preclinical models of EOC. Recent investigation reported that inhibition of c-MET enhances the activity of therapeutic drugs, including cisplatin^18^,^30^ and paclitaxel^31^,^32^,^33^ in EOC which suggested that c-MET mediated apoptotic resistance to conventional anticancer agents through the activation of a phosphatidylinositol 3-kinase (PI3K)/AKT-dependent pathway^18^,^31^. However, since most tumor specimens and tumor cell lines used in these prior studies have been ovarian serous adenocarcinoma, data analyzing the role of c-MET in OCCC have been limited. In OCCC cell lines, this study showed that inhibition of c-MET by SU11274 reduced cell proliferation and enhanced apoptosis (Figs 2 and 3). In addition, treatment with SU11274 effectively decreased expression of the p-c-MET proteins and blocked the phosphorylation of down-stream proteins Akt and Erk (Fig. 4).

Tang *et al*. recently reported that c-MET inhibitors with multikinase activity like crizotinib may exhibit less activity in ovarian cancer than c-MET specific drugs^34^. SU11274 is a selective inhibitor of c-MET, which has been shown to suppress the proliferation of hepatocellular carcinoma cells and pancreatic cancer cells^35^,^36^. Firtina Karagonlar *et al*. reported that SU11274 reversed the increased migration and invasion ability of sorafenib-resistant hepatocellular carcinoma cells^37^. In ovarian cancer, *in vitro* study, demonstrated that SU11274 reduced cell growth, motility, and invasive activity of EOC cells^16^. Our data are consistent with the previous results that SU11274 influences cell proliferation and apoptosis in OCCC. Moreover, SU11274 significantly decreased tumor weight in an orthotopic ovarian cancer mouse model with RMG1 cells and also in a PDX model of OCCC compared with that of the control group (Figs 5 and 6).

We can consider several limitations in this study. First, this study was performed with relatively small number of OCCC cell lines for *in vitro* experiments. Second, the biological effects of HGF and c-MET interactions were not investigated. According to recent study, Kwon *et al*. reported that anti-proliferative activity of DCC-2701, c-MET inhibitor, was dependent on c-MET activation which is induced by stromal derived HGF, and lesser extent exogenous HGF. They suggested that post-translational modifications of growth factors, such as HGF, might be one of the mechanisms that promotes tumor growth via factors produced by the microenvironments^38^. Lastly, the interaction between c-MET inhibitor and any particular OCCC molecular features beside p-c-MET expression was not identified. Therefore, further investigations should be conducted to unravel the detailed mechanisms by which c-MET modulation could be therapeutic in OCCC.

In conclusion, we found that unique activation of c-MET in OCCC tissues. In addition, inhibition of c-MET with the small molecule c-MET inhibitor (SU11274) showed potent anti-tumor activity in OCCC using *in vitro* and *in vivo* experiments. This work suggests that c-MET targeting therapy may be promising for OCCC treatment. Further clinical studies focused on c-MET inhibitors in OCCC are warranted.

## Materials and Methods

### Patients and tissue specimens

This study was reviewed and approved by the Institutional Review Board’s approval at Samsung Medical Center, Seoul, Korea (IRB # 2012-09-045). All experiments were performed in accordance with relevant guidelines. Written informed consent was obtained from each patient who participated in the investigation. Study subjects were enrolled from the Department of Obstetrics and Gynecology at Samsung Medical Center from July 2003 to November 2010. A total of 79 tissue specimens were collected including 16 normal ovarian tissues, 47 serous cancer tissues, and 16 clear-cell cancer tissues. Normal ovarian tissues were obtained from patients who underwent hysterectomy for benign disease, and tumor tissues were obtained from patients with ovarian cancer during surgery. All specimens were immediately snap frozen at −80 °C. The specimens were stained with hematoxylin and eosin (H&E) and evaluated by a gynecologic pathologist (ME Hong). Specimens with more than 90% tumor cells were used in the analysis. Histological typing of each tumor was conducted in accordance with the World Health Organization guidelines.

### RNA extraction and real-time RT-PCR

Total RNA, including mRNA, was extracted from the tissues using the mirVana miRNA isolation kit (Ambion, Texas, USA). cDNA was synthesized from total RNA using oligo dT primer (Invitrogen, San Diego, USA) in accordance with the manufacturer’s TaqMan RNA assay protocol (PE Applied Biosystems, San Francisco, USA). Real-time PCR was performed using an Applied Biosystems 7900HT Sequence Detection system (Applied Biosystems) in accordance with the manufacturer’s protocol. Data normalization was performed using GAPDH. To avoid amplification of genomic DNA, the primers and probes for amplifying c-MET (Met) and GAPDH were chosen to hybridize at the junction between the 2 exons as follows: c-MET (Met) (Hs1565584_m1; Applied Biosystems) and GAPDH (#4310884E; Applied Biosystems). Relative quantification of c-MET expression was calculated using the 2^−ΔΔC^_T_ method.

### Cell lines and treatments

ES2 was obtained from the American Type Culture Collection (ATCC, Manassas, VA, USA) and RMG1 and RMG2 were purchased from Japan Health Science Research Resources Bank (HSRRB, Osaka, JAPAN). A2780 was purchased from the European Collection of Cell Cultures (ECACC, Cat NO.93112520). SKOV3ip1, HeyA8, A2780-CP20, HeyA8-MDR and SKOV3-TR were a gift from Dr. Anil K. Sood, Department of Cancer Biology, University of Texas M.D. Anderson Cancer Center, TX, USA. ES2, RMG1 and RMG2 cells were originally derived from ovarian clear cell carcinomas (OCCCs); the other cell lines are non-OCCC cells^39^. Human EOC cell lines were maintained in complete media (A2780, A2780-CP20, SKOV3ip1 and SKOV3-TR: RPMI 1640, ES-2: McCoy’s 5 A, RMG-1 and RMG-2: Ham’s F12) supplemented with 10% fetal bovine serum (FBS) and 0.1% gentamicin sulfate (Gemini Bioproducts, Calabasas, USA) in 5% CO_2_ at 37 °C. The c-MET kinase inhibitors, cirzotinib (3-[(1 R)-1-(2,6-dichloro-3-fluorophenyl)ethoxy]-5-(1-piperidin-4-ylpyrazol-4-yl)pyridine-2-amine) (Cayman chemical, Item NO.12087) and SU11274 ((3Z)-N-(3-Chlorophenyl)-3-[[3,5-dimethyl-4-(4-methylpiperazine-1-carbonyl)-1H-pyrrol-2-yl]methylidene]-N-methyl-2-oxo-1H-indole-5-sulfonamide) (Calbiochem, Item NO. 448101), were resuspended in dimethyl sulfoxide (DMSO) at a concentration of 100 μg/ml. Cells were seeded at 3 × 10^3^ cells/well in 96-well microplates in culture medium with 10% fetal bovine serum (FBS). Cells were treated with inhibitor based on previous reports^40^.

### Western blot analysis

Cells or tissues were lysed in PRO-PRE Protein Extraction Solution (Intron Biotechnology, Seongnam, Korea). Whole cell lysates were prepared from tumor cell lines or tumor tissues and cells were lysed with lysis buffer containing 1 mM EDTA, 1% TX-100, 100 mM NaCl, 50 mM NaF, TrisHCl pH 7. 5, phosphatase inhibitor cocktail (Sigma-Ald rich, St. Louis, MO). Protein concentration was determined by Bradford assay kit (BIO-RAD, Hercules, USA). Cell or tissue lysates (40 μg of total protein) were separated in 8% acrylamide gels by sodium dodecyl sulfate-polyacrylamide gel electrophoresis (SDS-PAGE) and transferred to Hybond-ECL nitrocellulose filter paper (Amersham Biosciences, Buckinghamshire, UK). Membranes were blocked with 5% skim milk in Tris-buffered saline containing 0.1% Tween-20 for 1 hour at room temperature. Protein bands were probed with antibodies against c-MET and phospho-c-MET (p-c-MET) at a 1:200 dilution (Abcam ab74217 & ab5662), or total-Akt, phospho-Akt (p-Akt), total-ERK, phospho-ERK (p-ERK) (Cell Signaling Technology), and β-actin at 1:3000 (Santa Cruz, USA), and then labeled with horseradish peroxidase-conjugated anti-rabbit antibody (GE Healthcare, Piscataway, USA). Bands were visualized by enhanced chemiluminescence using an ECL kit (Amersham Biosciences, Buckinghamshire, UK) in accordance with the manufacturer’s protocol.

### 3-(4, 5-dimethylthiazol-2-yl)-2, 5-diphenyl tetrazolium bromide (MTT) assay

The MTT assay is based on the conversion of MTT to insoluble MTT-formazan by cleavage of the tetrazolium ring by mitochondrial dehydrogenase enzymes of living cells. Cells were treated with 1, 2, 3 and 5 μM of SU11274 and 0.1, 0.5, 1 and 2 μM of crizotinib for 48 h and 72 h, respectively. MTT solution (Amresco, Solon, USA) was added to each well. After 4 hours of incubation, the medium was discarded, and 100 μl of acidic isopropanol (0.1 N HCl in absolute isopropanol) was added and the plate gently shaken. Absorbance was measured on an enzyme-linked immunosorbant assay (ELISA) reader at a test wavelength of 540 nm. Each sample was assayed in triplicate and the experiment was repeated three times.

### Apoptosis assay

The relative percentage of apoptotic cells was assessed at 48 hours using the Annexin V-FITC Apoptosis Detection Kit- 1 (BD Pharmingen, San Diego, CA, USA) in accordance with the manufacturer’s protocol. Briefly, cells were washed twice in PBS and treated with SU11274 or crizotinib. The pellet was re-suspended in annexin V binding buffer at a concentration of 10^6^ cells/mL. Annexin V FITC and propidium iodide (PI) were added (5 μL per 10^5^ cells). Samples were gently mixed and incubated for 15 mins at room temperature in the dark before fluorescence activated cell sorter (FACS) analysis. Cell apoptosis was analyzed by staining Annexin V with using flow cytometry after 48 h of 5 μM of SU11274 or 0.5 μM of crizotinib incubation. Both early apoptotic (annexin V +, propidium iodide −) and late apoptotic (annexin V +, propidium iodide +) cells were defined as apoptosis.

### Immunohistochemical analysis

The primary antibodies used were against p-c-MET (Abcam ab5662). Tissue sections were deparaffinized three times in xylene for a total of 15 mins and subsequently rehydrated. Immunostaining for p-c-MET was performed using a Bond-maxTM automated immunostainer (Leica Biosystems, Melbourne, Australia) and the BondTM Polymer Refines Detection kit (Vision Biosystems, Melbourne, Australia). Briefly, antigen retrieval was carried out at 97 °C for 20 mins in ER1 buffer. After blocking the endogenous peroxidase activity with 3% hydrogen peroxidase for 10 mins, primary antibody incubation was carried out for 15 mins at room temperature at an antibody dilution of 1:200. Negative controls (substitution of primary antibody for TBS) were run simultaneously. Immunohistochemical staining for Ki67 was done as described previously^20^. Apoptotic positive cells were analyzed by TUNEL assay using the DeadEnd colormetric TUNEL assay kit (Promega, Madison, WI, country) as described previously^41^.

### Enzyme-linked immunosorbent assay (ELISA)

For the analysis of caspase-3, tumor tissues were lysed with lysis buffer containing 1 mM EDTA, 1% TX-100, 100 mM NaCl, 50 mM NaF, Tris-HCl ph 7.5, phosphatase inhibitor cocktail (sigma-Aldrich). Lysates were clarified at 13000 rpm for 20 min at 4 °C. Total lysates samples were assayed for concentrations of caspase-3 by using a ELISA kits. ELISA kits were used as described by the manufacturer to measure concentrations of human caspase-3 (R&D Systems, Abingdon, UK) The samples were measured in triplicate.

### Animal care and development of *in vivo* models including established cell line and PDX

Female BALB/c nude mice were purchased from Orient Bio, Seongnam, Korea. This study was reviewed and approved by the Institutional Animal Care and Use Committee (IACUC) of the Samsung Biomedical Research Institute (protocol No. H-A9-003) which is a facility accredited by the Association for the Assessment and Accreditation of Laboratory Animal Care International (AAALAC International) that performs in accordance with the Institute of Laboratory Animal Resources (ILAR) guide. All methods were carried out in accordance with the relevant guidelines. To establish orthotopic models, RMG1 (5 × 10^6^ cells/0.2 mL HBSS) cells were injected into the peritoneal cavity of mice^42^. For the PDX model of OCCC, patient tumor specimens retrieved from operating room were sliced into small pieces (below 2–3 mm), implanted into the left subrenal capsule of mice^24^ and propagated by serial transplantation. Depending on tumor tissue availability, tumor fragments were implanted in a minimum of five mice. The mice used in these experiments were 6 to 8 weeks old. Mice (*n* = 10 per group) were monitored daily for tumor development and sacrificed either on day 70–90 after the injection of cancer cells (orthotopic RMG1 model), or on day 60–70 (PDX model) or when any of the mice seemed moribund. We recorded total body weight, tumor weight and the number of tumor nodules. Tumors were fixed in formalin and embedded in paraffin or snap frozen in OCT compound (Sakura Finetek Japan, Tokyo, Japan) in liquid nitrogen.

### Data analysis

Each experiment was carried out at least three times in triplicate. The results are presented as the means ± standard deviation (SD) of three independent experiments. The Mann–Whitney *U* test was used to compare differences among groups for both *in vitro* and *in vivo* assays. SPSS software (version 17.0; SPSS, Chicago, USA) was used for all statistical analyses. All statistical tests were two-sided and *p* values less than 0.05 were considered to be statistically significant.

## Additional Information

**How to cite this article**: Kim, H.-J. *et al*. c-MET as a Potential Therapeutic Target in Ovarian Clear Cell Carcinoma. *Sci. Rep.* **6**, 38502; doi: 10.1038/srep38502 (2016).

**Publisher's note:** Springer Nature remains neutral with regard to jurisdictional claims in published maps and institutional affiliations.

## Supplementary Material

Supplementary Information
