# Supplementary material for: c-MET as a Potential Therapeutic Target in Ovarian Clear Cell Carcinoma
Source: Sci Rep. 2016 Dec 5;6:38502. doi: 10.1038/srep38502 (PMC5137074; doi:10.1038/srep38502)
Supplement: Supplementary Information [file srep38502-s1.doc]

**c-MET as a Potential Therapeutic Target in Ovarian Clear Cell Carcinoma**

Ha-Jeong Kim^1^,*, Aera yoon^2^,*****, Ji-Yoon Ryu^2^,*****, Young-Jae Cho^2^, Jung-Joo Choi^2^, Sang Yong Song^3^, Heejin Bang^3^, Ji Soo Lee^4^, William Chi Cho^5^, Chel Hun Choi^2^, Jeong-Won Lee^2,6,7†^, Byoung-Gie Kim^2^, and Duk-Soo Bae^2^

^1^Department of Obstetrics and Gynecology, Institute of Wonkwang Medical Science, College of Medicine, Wonkwang University, Iksan, Korea; ^2^Department of Obstetrics and Gynecology, ^3^Department of Pathology and Translational Genomics, ^4^Health promotion center Samsung Medical Center, Sungkyunkwan University School of Medicine, Seoul, Korea. ^5^Department of Clinical Oncology, Queen Elizabeth Hospital, Kowloon, Hong Kong. ^6^Institute for Refractory Cancer Research, Samsung Medical Center, Seoul, Korea. ^7^Samsung Advanced Institute for Health Sciences & Technology, Sungkyunkwan University School of Medicine, Seoul, Korea.

*****These authors contributed equally to this work.

**Corresponding Author: Jeong-Won Lee MD, PhD.**

Department of Obstetrics and Gynecology, Samsung Medical Center, Sungkyunkwan University School of Medicine, 81 Irwon-ro, Gangnam-gu, Seoul 135-710, Korea. Tel.: +82 2 3410 1382; fax: +82 2 3410 0630. E-mail: garden.lee@samsung.com


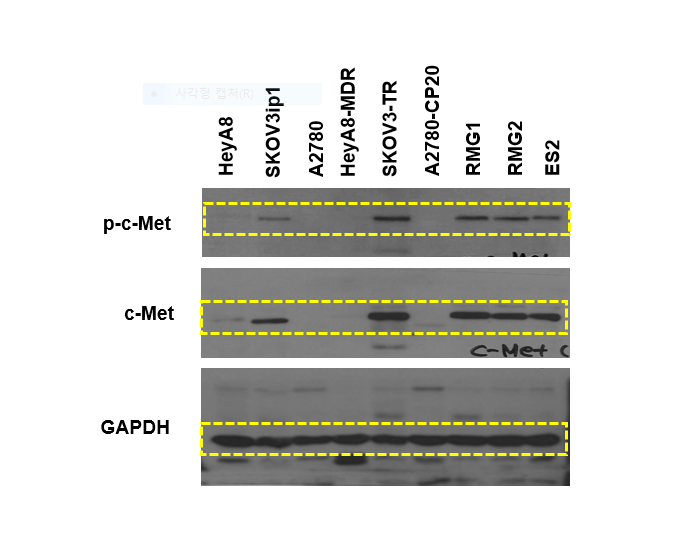


**Supplementary Figure 1.** Expression of c-MET in ovarian cancer cell lines measured using Western blot.


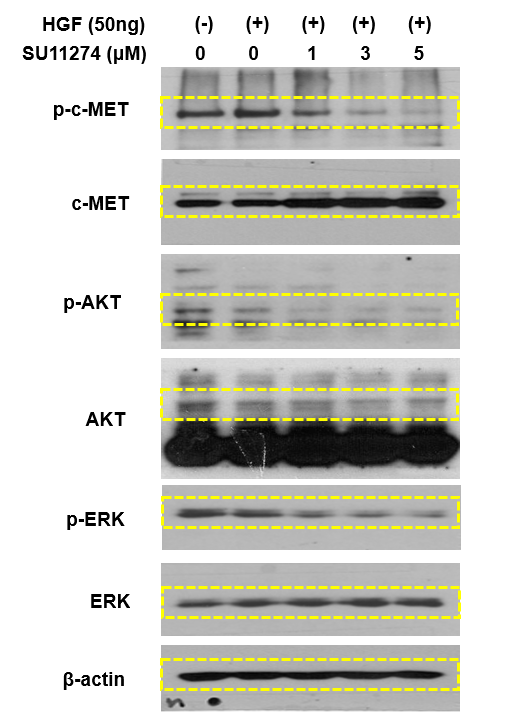


**Supplementary Figure 2.** SU11274 inhibited the expression of c-MET and downstream signaling proteins in ES2 cells. Based on Western blots, phospho-c-MET and phosphorylation of downstream signaling proteins including p-Akt and p-Erk were decreased by treatment with SU11274 in a dose-dependent manner.

**
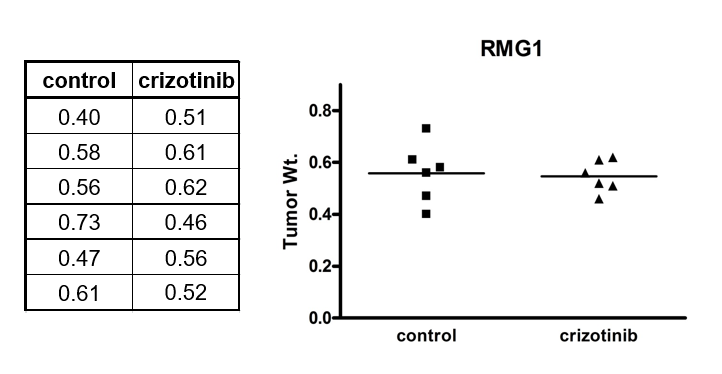
**

**Supplementary Figure 3.** Effect of crizotinib administration on tumor growth in RMG1 mouse model. There was no significant difference in reduction of tumor weight between crizotinib-treated group and untreated group ( p = 0.852).


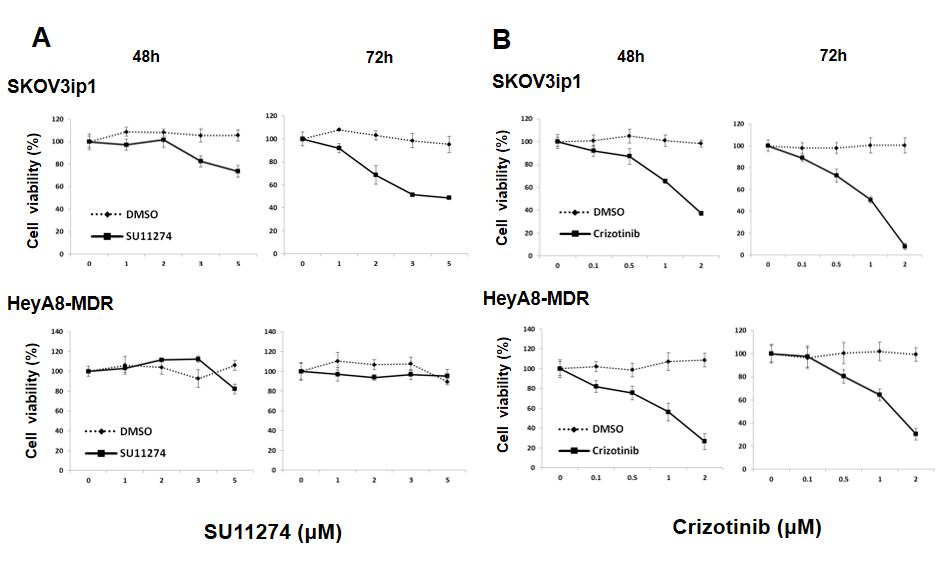


**Supplementary Figure 4.** Cell viability assay with SU11274 in SKOV3ip1 and HeyA8-MDR cells. A. SU11274 treatment was able to reduce cell viability in SKOV3ip1 but not in HeyA8-MDR cells. However, the effect of SU11274 on cell viability inhibition was greater in OCCC cells with high c-MET expression than in SKOV3ip1 or HeyA8-MDR cells with low c-MET expression. B. Crizotinib also reduced cell viability in SKOV3ip1 and HeyA8-MDR cells. However, it is may be due to its strong activity against anaplastic lymphoma kinase (ALK).

**
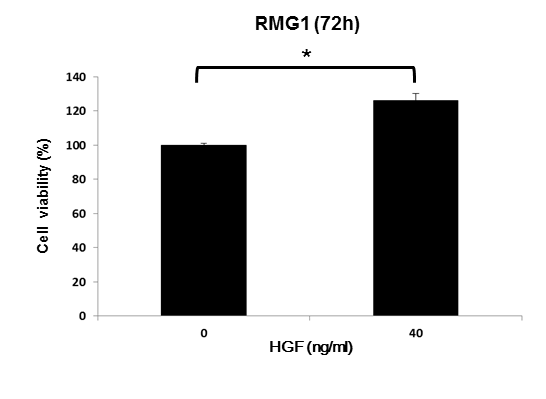
**

**Supplementary Figure 5.** Cell viability assay with HGF in RMG1 cell. RMG1 cell was treated with 40ng HGF for 72hr. HGF increased cell viability about 20% in RMG1 cell line compared with control (p = 0.049)


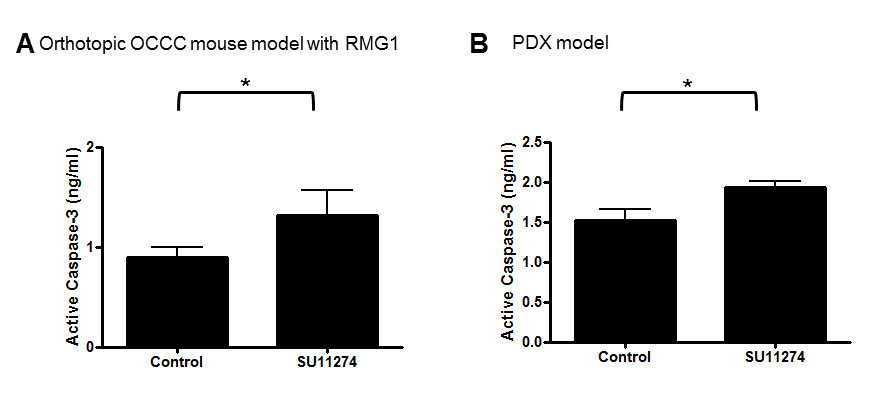


**Supplementary Figure 6.** Quantitative representation of caspase-3 activity in RMG1 mouse model and PDX model. The treatment of 6mg/kg SU11274 significantly increased apoptosis compared with the control in RMG1 mouse model and PDX model (p = 0.049 and p = 0.026).
